# Comment on ‘Detection of Cancer‐Associated Cachexia in Lung Cancer Patients Using Whole‐Body [^18^F]FDG‐PET/CT Imaging: A Multicentre Study’ by Ferrara et al.

**DOI:** 10.1002/jcsm.13656

**Published:** 2024-11-06

**Authors:** Hao Chen, Xiangyu Shen, Xiaodong Chen

**Affiliations:** ^1^ Department of Gastrointestinal Surgery, the First Affiliated Hospital Wenzhou Medical University Wenzhou China

To the Editor

We read with great interest the recently published article by Ferrara et al. [1] in the *Journal of Cachexia, Sarcopenia and Muscle*. Based on whole‐body [^18^F]FDG‐PET/CT imaging, the study demonstrated the groupwise differences in the multi‐organ metabolism of lung cancer patients (LCP) with and without cancer‐associated cachexia (CAC), thus highlighting systemic metabolic aberrations symptomatic of cachectic patients and identifying LCP with CAC accurately by machine‐learning model. We commend the authors for their valuable contributions and offer several suggestions which could further enhance the interpretation of these findings and provide valuable direction for future research.

Firstly, some detailed covariates related to comorbidities and lifestyle factors may not have been fully considered in the baseline analysis. Cachexia is a multifactorial syndrome influenced by cancer itself as well as comorbidities and lifestyle factors, including smoking, alcohol use and dietary pattern. Including the detailed covariates and performing subgroup analyses would provide more granular insights into how these variables interact with cachexia. Considering these factors can potentially reveal which subgroups may benefit the most from this analysis and further enhance the robustness of the study results.

Secondly, the assessment of CAC may be biassed. The study utilises the Weight Loss Grading System (WLGS) to assess cachexia, which classifies patients based on BMI and weight loss over 6 months [2]. There are concerns regarding its reliance on self‐reported weight loss data and the lack of standardised weight measurements, which introduces the risk of recall bias. In future studies, incorporating more standardised weight measurement techniques, as well as functional assessments like grip strength or gait speed, could reduce bias and enhance the reliability of cachexia evaluation. Additionally, surgical resection is a common treatment in LCP, removing lesions in the lungs also causes varying degrees of weight loss. While the study has excluded WLGS 2 patients to minimise non–cachexia‐related weight loss, they do not provide enough details regarding the type of treatment the study participants received during the study period. To address this, it would be beneficial to specify the types of treatments the patients underwent and assess their impact on weight loss separately from cachexia.

Thirdly, the imaging approach used in the study primarily focuses on the metabolic activity of solid organs, but it overlooks the imaging features from hollow organs and key tumour‐related factors that could provide additional insights into cachexia. The tumour microenvironment, including both intratumoral and peritumoral regions, plays a critical role in systemic inflammation and metabolic dysregulation [3, 4]. Moreover, the hollow organs like the gastrointestinal tract are directly involved in digestion and nutrient absorption, both of which are crucial to energy homeostasis. It is surprising that the authors did not extract or analyse these imaging characteristics in their assessment. Future research should incorporate a more comprehensive extraction of imaging features to better understand the multifaceted mechanisms of CAC.

In conclusion, we greatly appreciate the valuable insights this article provides on the association between CAC in LCPs and whole‐body [^18^F]FDG‐PET/CT imaging. We look forward to future research that further explores and addresses these limitations, inspiring further discussion and more comprehensive evaluations in CAC research.

## Conflicts of Interest

The authors declare no conflicts of interest.
